# Tubridge flow diverter for the treatment of dissecting aneurysms in the middle cerebral artery

**DOI:** 10.3389/fneur.2025.1552610

**Published:** 2025-03-27

**Authors:** Yu Duan, Guohui Huang, Jun Shen, Ziwei Xu, Zhuyu Li, Jian Li, Dongwei Dai

**Affiliations:** ^1^Department of Neurosurgery, Huadong Hospital, Fudan University, Shanghai, China; ^2^Department of Neurovascular Center, Changhai Hospital, Naval Medical University, Shanghai, China; ^3^Department of Neurology, Huadong Hospital, Fudan University, Shanghai, China

**Keywords:** dissecting aneurysms, flow diverter, middle cerebral artery, branches, stent

## Abstract

**Background:**

The Tubridge flow diverter (TFD) has become a widely used device for reconstructing parent vessels and sealing complicated aneurysms in China. However, its application to managing distal small to medium-sized dissecting aneurysms has not been extensively investigated. This study aimed to evaluate the safety and effectiveness of the TFD and the factors affecting healing in patients with dissecting aneurysms in the middle cerebral artery (MCA).

**Methods:**

Patients with dissecting aneurysms in the MCA who were treated with the TFD from 2019 to 2023 were included. According to the O’Kelly–Marotta (OKM) scale, OKM grade A was defined as dense embolism, while OKM grades B to D were defined as non-dense embolism in this study. Clinical information, the degree of aneurysm occlusion, and clinical outcomes were retrospectively analyzed.

**Results:**

A total of 25 patients with 25 MCA dissecting aneurysms were identified. The average age was 52.4 years (age range 20–76), with 5 patients (20%) experiencing subarachnoid hemorrhage. In total, 12 aneurysms were located in the M1 segment, 11 involved the M2 bifurcation, and two were in the M3 segment or above. The median aneurysm length was 17.3 mm (range 4.2–27), and the average width was 5.4 mm (range 2.3–7.6). A total of 12 cases had one or more branches in the MCA. Furthermore, four cases (16%) showed asymptomatic in-stent stenosis, and five cases (20%) had main branch injuries during the angiographic follow-up. A total of three patients experienced acute ischemic events, and one had not fully recovered during the follow-up. There were no deaths related to procedure complications. According to the single-factor analysis, the patients in the non-dense embolism group during the follow-up had more strong branches before the operation (χ^2^ = 9.001, *p*=0.003).

**Conclusion:**

The TFD is a safe and effective flow diverter for distal dissecting MCA aneurysms. The aneurysms with strong branches were associated with a higher rate of non-dense embolism during the follow-up.

## Introduction

As we know, it remains challenging to manage dissecting aneurysms in the middle cerebral artery (MCA) with complex anatomies, particularly those with wide-neck configurations that involve small, critical branches (1, 2). In the past, many neurosurgeons preferred clipping, wrapping, or ligating MCA dissecting aneurysms as the primary treatment option (3–5). Recently, flow diverters (FDs) have been regarded as a promising treatment option for treating complex and distorted wide-neck aneurysms, including those with many small branches. However, the efficacy and safety of this technique are not yet fully understood (6, 7). The Tubridge flow diverter (TFD, MicroPort Neurotech, Shanghai, China), an innovative self-expanding stent made of nickel-titanium braiding, has been proven to be a feasible and effective treatment for complex, large, and giant intracranial aneurysms in China (8, 9). It could also be applied to small internal carotid artery (ICA) (10) dissecting aneurysms (11, 12).

In this study, we conducted a retrospective analysis focusing on the treatment of MCA dissecting aneurysms using the TFD, with the goal of elucidating the following aspects: (1) the occlusion rate and its relevant factors, (2) treatment-related complications and clinical outcomes, and (3) the outcome for small branches after TFD deployment.

## Methods

### Patient selection

The study was approved by our institutional review board, and all patients provided general informed consent. We reviewed our database to identify dissecting aneurysms in the MCA treated with flow diversion between May 2019 and May 2023.

The inclusion criteria were as follows: (1) Dissecting aneurysms located in the middle cerebral artery, as identified using cerebral digital subtraction angiography (DSA) and high-definition magnetic resonance imaging; (2) patients over 18 years old; and (3) treatment with TFD. The exclusion criteria were as follows: (1) aneurysms associated with trauma, mycosis or infection, and inflammation, and (2) previous surgical clipping or stent-assisted coiling.

Based on the Sacho’ study, dissecting aneurysms were defined as those with an aneurysmal dilation exceeding 50% of the vessel wall circumference (13). The aneurysms were classified into three types: (1) located in the M1 segment only; (2) involving the M2 bifurcation; and (3) located in the distal MCA, from M3 to M4. Small cortical branches arising before the division of the MCA into a superior and inferior trunk, or three divisions, and their relationship with the aneurysm were noted.

Clinical data on patient demographics, symptoms at presentation, postoperative angiograms, complications, clinical outcomes, and follow-up imaging were all collected (see Table 1).

**Table 1:** Clinical information of the 25 patients and comparison between the dense embolism group and non-embolism group.

	Total patients	Dense embolism group	Non-embolism group	Test value	*P*-value
Age (years)	52.4 ± 15.1	53.1 ± 14.3	51.5 ± 16.8	0.272^a^	0.788
Male (*n*, %)	19 (76.0%)	11 (78.6%)	8 (72.7%)	0.115^b^	0.734
Location (*n*, %)				0.065^b^	0.968
M1	12 (48.0%)	7 (50.0%)	5 (45.5%)		
M2	11 (44.0%)	6 (42.9%)	5 (45.5%)		
M3 or above	2 (8.0%)	1 (7.1%)	1 (9.1%)		
Maximum length (mm)	17.3 (7.6)	17.7 (6.5)	16.1 (6.2)	−1.369^c^	0.171
Maximum width (mm)	5.4 ± 1.5	5.7 ± 1.5	5.3 ± 1.5	0.667^a^	0.511
Pre-rupture	5 (20%)	4 (28.6%)	1 (9.1%)	1.461^b^	0.227
Pre-recurrent	3 (12.0%)	5 (35.7%)	0	2.679^b^	0.102
With branches	12 (48.0%)	3 (21.4%)	9 (81.8%)	9.001^b^	0.003
Pre-mRS				3.071^b^	0.215
0	19 (76.0%)	9 (64.3%)	10 (90.9%)		
1	3 (12.0%)	2 (14.3%)	1 (9.1%)		
2	3 (12.0%)	3 (21.4%)	0		
In-stent stenosis	4 (16.0%)	1 (7.1%)	3 (27.3%)	1.857^b^	0.173
Injured side branches	5 (20%)	2 (14.3%)	3 (27.3%)	0.649^b^	0.421
Acute ischemic events	3 (12.0%)	1 (7.1%)	2 (18.2%)	0.711	0.399
Follow-up				2.679^b^	0.262
No change	20 (80.0%)	11 (78.6%)	11 (100%)		
Improvement	2 (8%)	2 (14.3%)	0		
Deterioration	1 (12.0%)	1 (7.1%)	0		

The normally distributed measurement data are presented as mean and standard deviation, while the non-normally distributed measurement data are presented as median and quartile spacing.

a T value.

b χ^2^ value.

c Z value.

### Antiplatelet agents

The patients received two dual antiplatelet therapy regimens: 100 mg/d aspirin and 75 mg/d clopidogrel (from March 2019 to February 2021), and 100 mg/d aspirin and 90 mg 90 mg/bid ticagrelor (from March 2021 to March 2023). The dual antiplatelet therapy was carried out 5 days before the procedure and continued for 3 months. The monotherapy of aspirin was continued for the next 9 months.

### Endovascular treatment

All procedures were performed under general anesthesia. Systemic heparinization was administered following sheath placement to prevent blood clotting during the procedure. An 8F femoral artery sheath with a 6F long sheath (80 or 90 cm) was used. After the long sheath reached the origin of the common carotid artery (CCA), an intracranial support catheter was navigated to the distal internal carotid artery. The Fastrack microcatheter was advanced over a 0.014-inch guidewire into the M3 segment or beyond in preparation for flow diverter delivery. The appropriate size and length of the TFD were selected based on the diameter of the parent artery and the length of the aneurysm. Under fluoroscopic guidance, for patients treated with adjunctive coil embolization, an Echelon−10 microcatheter (Medtronic, Minneapolis, MN, United States) was advanced into the aneurysm via contralateral femoral artery access for coiling after the implantation of the flow diverter.

### Evaluations and follow-up

Clinical evaluation took place immediately after each procedure, as well as at discharge and 3-, 6-, and 12-months post-procedure. The modified Rankin Scale (mRS) was used to assess clinical outcomes (14). Morbidity and mortality were defined as any deterioration in the mRS after the procedure and death related to treatment, respectively. CT angiography was usually performed 3 months after the procedure. Subsequent angiographies were performed initially at 6 months and then again at 12 months during the follow-up. Aneurysm occlusion was evaluated based on the O’Kelly–Marotta (OKM) scale: A—total filling, B—subtotal filling, C—entry remnant, and D—no filling (15). In this study, OKM grade A was defined as dense embolism, while OKM grades B to D were defined as non-dense embolism. The jailed side branch by the TFD was described as follows: (1) patent (unchanged), (2) narrowing of the diameter (stenosed) or orifice, and (3) occluded.

### Statistical analysis

The data were analyzed using Statistical Package for the Social Sciences Version 25.0 for Windows (SPSS, Chicago, Illinois, United States). Normally distributed data were presented as mean and standard deviation and analyzed using the independent samples *t*-test. Non-normally distributed data were presented as median and quartile spacing and analyzed using the Mann–Whitney U-test. Categorical variables were presented as percentages and analyzed using Pearson’s χ^2^ test. A *p*-value of <0.05 was considered statistically significant.

## Results

### Patient baseline and aneurysm characteristics

**Figure 1:**
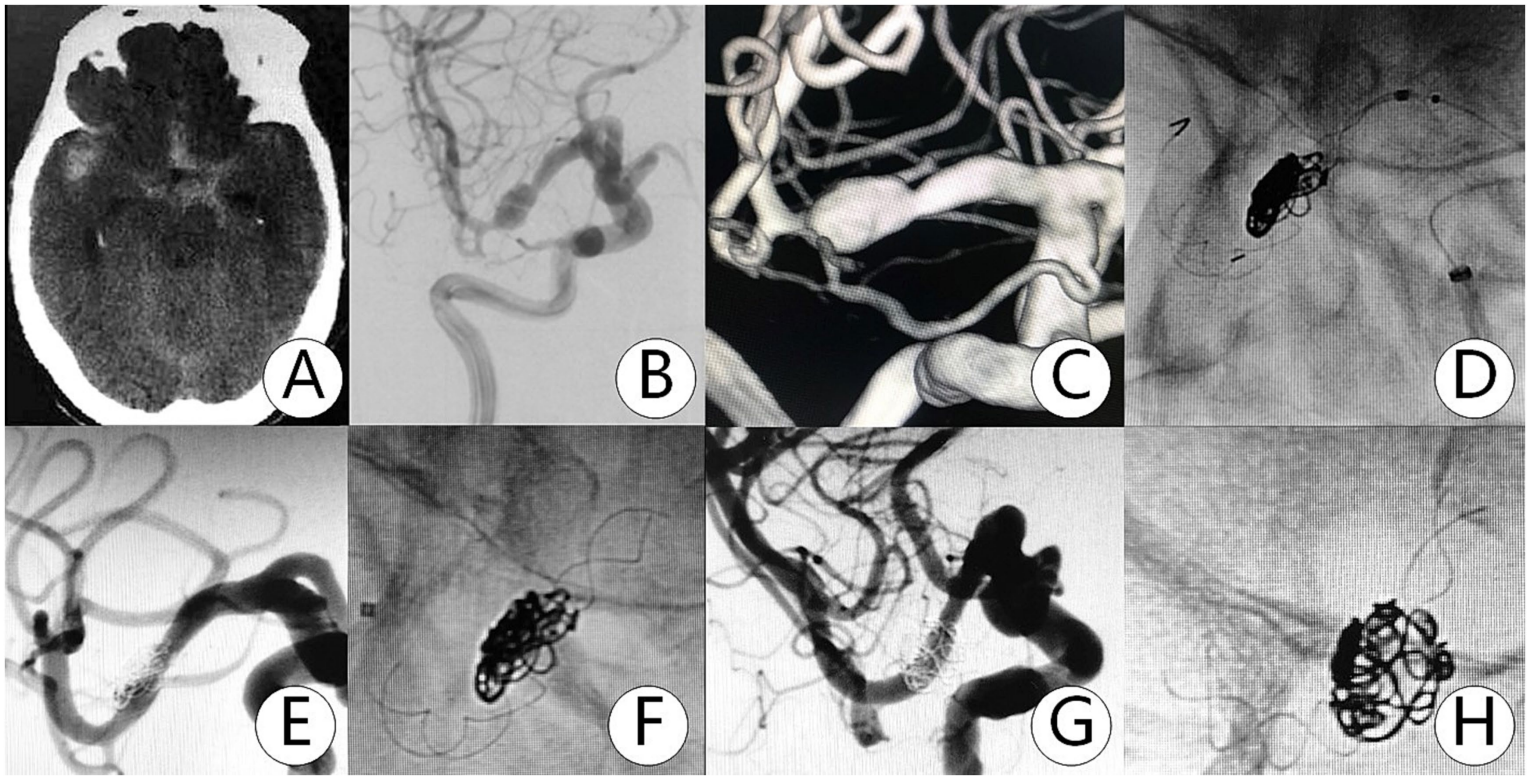
Patient experienced a subarachnoid hemorrhage with a Hunt–Hess grade I for 5 h. **(A)** CT showed subarachnoid hemorrhage in the Sylvian cistern and pericallosal cistern. **(B,C)** DSA revealed a dissecting aneurysm in the M1 segment. **(D–F)** The TFD (3.0×25 mm) and coils were successfully deployed in the aneurysm. **(G,H)** DSA at 13 months follow-up showed that the dissecting aneurysm was completely embolized.

**Figure 2:**
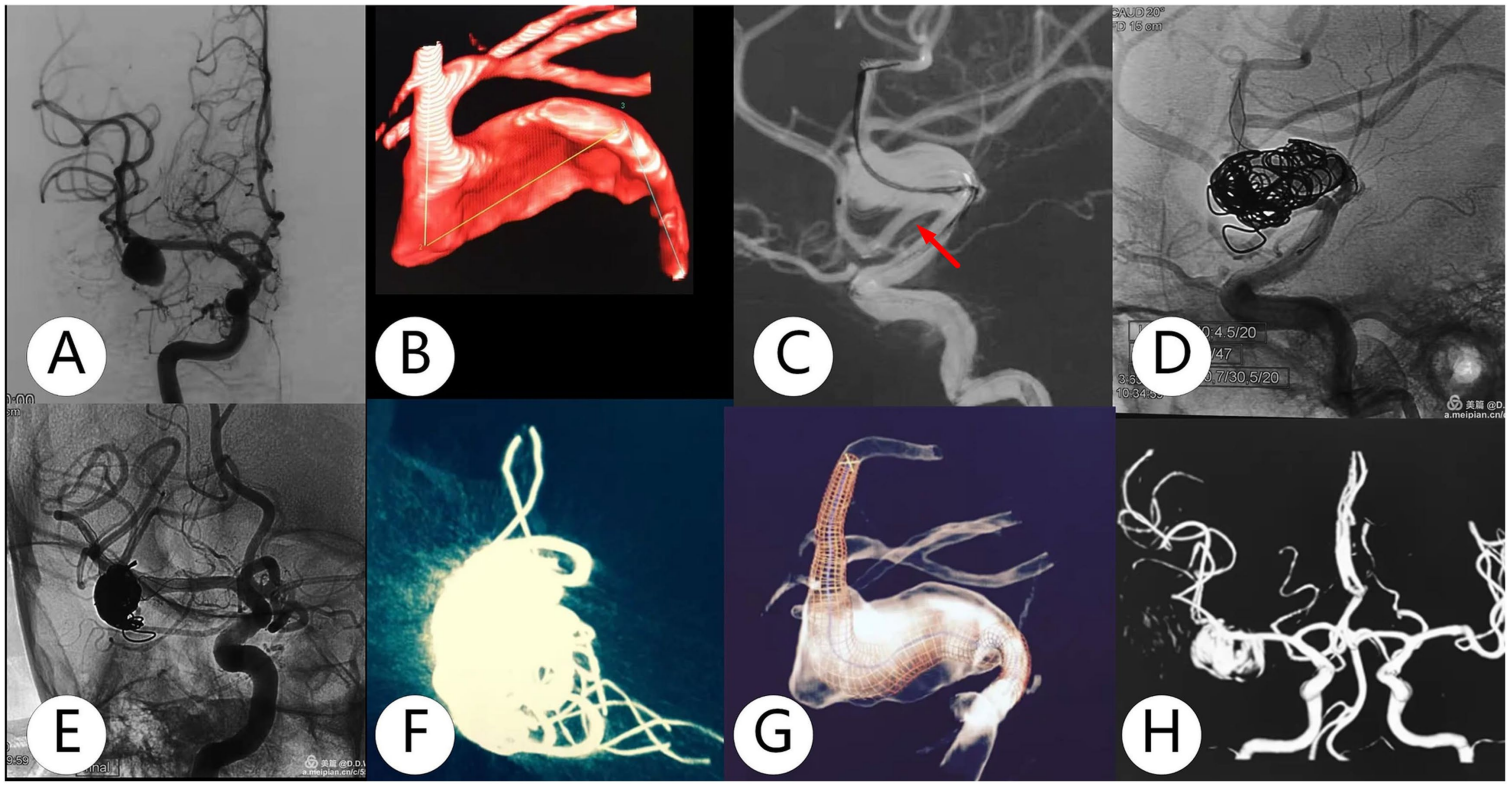
Adult was diagnosed with a right dissecting aneurysm during a health check. **(A,B)** The dissecting aneurysm extended from M1 to M2 with a strong branch. **(C,D)** The TFD (3.0×30 mm) with coils was deployed. **(E–G)** The reconstruction showed that the TFD unfolded well. **(H)**. MRA at 1-year follow-up showed that the dissecting aneurysm remained at O’Kelly–Marotta grade C.

A total of 25 cases of dissecting aneurysms in the MCA treated with the TFD were included. The average age was 52.4 years (age range 20 ~ 76), and 19 patients (76.0%) were female. The aneurysms were found incidentally in nine patients (36.0%), while five patients (20%) experienced hemorrhage due to the rupture of the aneurysm (Figure 1). The remaining patients exhibited symptoms such as headache and dizziness (Figure 2).

In total, 12 dissecting aneurysms (48%) were in the M1 segment, 11 (44%) involved the M2 bifurcation, and 2 (8%) were in the M3 or above. The median aneurysm length was 17.3 mm (range 4.2–27), and the average width was 5.4 mm (range 2.3–7.6). In addition, 12 (48%) cases had one or more branches in the MCA segment.

A total of 10 (40%) cases were treated with the TFD assisted by coils, including all five ruptured dissecting aneurysms. The remaining 15 cases (60%) were treated with the TFD alone, and 2 cases (80%) were treated with 2 TFD stents deployed tandemly.

The immediate postoperative angiographic outcome showed that 4 cases (16%) of ruptured dissecting aneurysms were completely occluded (OKM A), 4 (16%) cases were assessed as OKM grade B (near complete occlusion), and 12 (48%) and 5 (20%) cases were assessed as OKM C and OKM D, respectively.

### Angiographic outcomes during the follow-up

During the angiographic follow-up (13-month median), 14 cases (44%) showed complete occlusion (OKM A) and were in the dense embolism group. A total of 11 cases (56%) were in the non-dense embolism group. Of these, eight (32%) were in OKM grade B (near complete occlusion), three (12%) were in OKM C, and there were no cases in OKM D. In total, 22 cases (88%) showed progress according to the embolization assessment during the follow-up. From another point of view, the rate of complete (OKM A) and near-complete (OKM B) occlusion reached 88%.

Differences in age, aneurysm size, aneurysm location, history of subarachnoid hemorrhage, complications, and prognosis were not statistically significant between the dense embolism group and the non-dense embolism group. According to the single-factor analysis, the patients in the non-dense embolism group had a higher rate of having branches in the MCA segment, as shown by preoperative DSA images (χ^2^ = 9.001, *p*=0.003).

### Treatment-related complications

A total of four cases (16%) showed asymptomatic in-stent stenosis, while five cases (20%) displayed branch injuries during the angiographic follow-up. Of those with branch injuries, one anterior cerebral artery (ACA) covered by the TFD showed narrowing, and four M1 to M2 branches covered by the TFD exhibited occlusion or a reduced caliber. In addition, three patients (12.0%) experienced procedure-related ischemic events during the follow-up. There were no deaths related to procedure complications.

A total of 24 patients (96%) had a good clinical outcome at the last follow-up (11 months to 36 months), and only 1 (4%) experienced a decline in neurological function from mRS 0 before the operation to mRS 1 at 23 months after the operation during the last follow-up.

## Discussion

Our study demonstrated that the TFD was also a safe and effective treatment for dissecting aneurysms in the MCA. The overall rate of complete occlusion (OKM A) and near complete (OKM B) was 88% (22/25), and 96% of the patients (24/25) received a good clinical outcome at the last follow-up. A total of 22 cases (88%) showed progress in embolization assessment during the follow-up. There were no cases of rupture or death. In the non-dense embolism group, the patients had a higher rate of having branches, which might have been an important factor contributing to incomplete healing during the follow-up.

### Safety and effectiveness

Despite similar clinical safety and efficacy in terms of obliteration rates and complications between the Pipeline embolization device (PED) and TFD (16, 17), the TFD, being made of nickel–titanium alloys, offers high shape-holding memory and super-elasticity. Its radiopaque microfilaments improve the visualization of the stent during deployment (15, 16).

A random-effects meta-analysis of FDs showed that the obliteration rate was satisfactory (>70%) and the risk of branch occlusion-related complications was low (incidence rate < 5%) after 1-year follow-up (18). Xie et al. (11) reported that the TFD showed satisfying results for vertebrobasilar artery dissecting aneurysms, with a complete occlusion rate of 78.26% and a mild asymptomatic cerebral infarction rate of 13.04%. However, there is still limited literature on the use of FDs for treating distal dissecting aneurysms in the MCA or above. Soydemir et al. (19) reported that in 76 MCA aneurysms treated with different FD stents, including the flow re-direction endoluminal device (FRED), FRED Jr., and PED, the branches were occluded in 10% of cases and/or 30.1% had in-stent stenosis, but none of the patients had neurological deterioration during follow-up. Gai’s et al. (12) study of the PED and TFD showed that the complete occlusion rate was 75% in eight cases of M1 dissecting aneurysms. However, 10% of the cases experienced procedure-related morbidity.

The risk of thromboembolic events caused by clopidogrel resistance is taken seriously (20), and since 2021, clopidogrel has been replaced by ticagrelor (21), in combination with aspirin, as the conventional antiplatelet regimen for the perioperative period at our center. There was no direct evidence that four patients with in-stent stenosis were associated with antiplatelet therapy in this cohort; however, two patients with in-stent stenosis were observed after ticagrelor was discontinued. For the small-vessel PED, we tend to apply assured higher-potency antiplatelet agents, such as ticagrelor, to decrease the risk of acute stent occlusion (22). Ge’s et al. (23) report indicated that a higher maximum platelet aggregation rate predicted higher rates of incomplete occlusion and in-stent stenosis and that the dose levels of ticagrelor showed no significant difference in clinical prognosis and aneurysm occlusion rates. Antiplatelet aggregation materials or medications coated on FDs, such as heparin, hydrophilic polymers, or others, will be promising therapeutic targets in the future (24, 25). We will further explore the associated risks and complications of FDs with the expansion of sample size and longer follow-up.

### Branches

In this cohort, 11 cases (44%) had one or more branches in the MCA segment, which is similar to the rate (66.6%) of Pervinder’ saccular aneurysms (26). It is admittedly recognized that MCA segment aneurysms with a deeper dissection have a close relationship with many small branches, leading to unpredictable outcomes. MCA aneurysms are readily accessible through an endovascular approach, and their morphology may make them suitable for flow diversion, either alone or with coil assistance (27, 28).

During the follow-up, we found that the branches hindered the dissecting aneurysm from achieving complete occlusion. The main reason was that continuous blood flow in the branches impeded stent reendothelialization. It was our basic concept that preserving the perforating vessels was more important than occluding the aneurysms itself. As we all know, perforating vessels in the MCA, such as lenticulostriate perforators, have irreplaceable functions, and if injured, they can lead to disastrous consequences. Bender reported a patient with a huge right MCA aneurysm who, after receiving the PED with adjunctive coiling, experienced a severe stroke. The injury to branches in the MCA was considered the pivotal cause of the stroke (27). Cagnazzo et al. (29) reported a thromboembolic complication rate of 16.3%, with symptomatic stroke related to jailed branch occlusion and slow flow occurring in approximately 5% of cases. In Gawltza’s et al. (30) study of FDs in the MCA bifurcation or anterior communicating artery complex, after cortical branches were covered, 41.2% of patients showed symptomatic or asymptomatic lesions in the perforator territories. In our cohort, we observed that five cases (20%) showed branch injuries, and three of them experienced mild stroke and hemiplegia after the operation. Based on our experience, if possible, it is important to avoid jailing the M2 branch (31), while ensuring adequate coverage of the aneurysm neck. We also agree that adjunctive coil embolization with a loose density (<12%) is necessary for large or giant dissecting aneurysms (32).

Our study has several limitations. First, the inherent bias associated with a retrospective, single-center design must be acknowledged. Second, the results from the subgroup analyses within this small population need to be confirmed through multi-center studies with larger sample sizes and longer follow-ups.

## Conclusion

The TFD is a safe and effective flow diverter for distal dissecting aneurysms in the MCA. The aneurysms with strong branches were associated with a higher rate of non-dense embolism during the follow-up.

## Data Availability

The raw data supporting the conclusions of this article will be made available by the authors, without undue reservation.
